# Advancements in Radiofrequency Ablation of Cardiac Arrhythmias Open New Possibilities for Procedural Safety

**DOI:** 10.19102/icrm.2020.111104

**Published:** 2020-11-15

**Authors:** Junaid Bhutto, Rahul N. Doshi

**Affiliations:** ^1^Cardiac Arrhythmia Group, Cardiovascular Center of Excellence, HonorHealth, Scottsdale, AZ, USA

**Keywords:** Atrial fibrillation, catheter ablation, low fluoroscopy, zero fluoroscopy

In this issue of the *Journal of Innovations in Cardiac Rhythm Management*, Zei et al. provide a comparison of the safety and efficacy of low- versus zero-fluoroscopy protocols for catheter ablation for atrial fibrillation (AF) using data from a multicenter, prospective registry.^1^ They report that no differences existed with respect to the primary outcomes of pulmonary isolation, safety, and procedural time between these two approaches. The data presented therefore provide food for thought—should we give up fluoroscopy completely?

The advent of both improved electroanatomical mapping (EAM) techniques and intracardiac echocardiographic (ICE) imaging has greatly improved our ability to achieve high-quality results in treating arrhythmia with or without the use of fluoroscopy.^2^ The frequency of fluoroscopy use has consistently decreased over the last decade of AF ablation.^3^ ICE imaging has helped to minimize risks of complications at the time of transseptal puncture and identify anatomic variations that are not visible under fluoroscopic views. Advances in EAM now also enable extremely detailed three-dimensional construction of cardiac chambers and the creation of an electrical map of these structures. While these techniques were developed to complement conventional fluoroscopic ablation procedures, they now offer an alternative route by which to achieve high-quality results without fluoroscopy. Zei et al. demonstrate comparable results between conventional techniques performed using low fluoroscopy and no fluoroscopy, respectively, with outcomes that are durable and without increased risk to the patients. This is valuable information for operators who still feel the need for some use of fluoroscopy, particularly for transseptal puncture.

The use of low or “very low” fluoroscopy has been widely adopted and large experiences have been reported it to be both safe and efficacious.^4^ These results have been reproduced by other investigators as well and provide us with the confidence to adopt these techniques when treating our patients. However, the use of “zero” fluoroscopy has been less well-studied. Razminia et al. previously reported their single-center experience with a spectrum of electrophysiology procedures with zero fluoroscopy.^5^ Haegeli et al. demonstrated the same in another single-center study.^6^ Some reports have also included cryoballoon procedures that have traditionally used venography to evaluate balloon occlusion and which instead employed ICE to assess flow around the balloon.^5^ These findings compel us to perhaps consider that a zero-fluoroscopic approach may be the preferred method of approaching ablations with the radiofrequency (RF) modality.

To make the case for any new approach, the scientific community must first consider its safety profile. The modality must also offer comparable or superior results to that of the conventional method. Furthermore, the modality must demonstrate additional advantages for it to supplant the conventional approach. Lastly, the limitations of the new modality must be evaluated as well to avoid its use in less-desirable scenarios.

The study in question reproduces the safety of using minimal fluoroscopic imaging that was previously demonstrated by Sommer et al.^2^ Zei et al. reproduced the results with zero fluoroscopy as well. The complication rates remained low and, more importantly, were not skewed to a complication that may suggest issues with the use of zero fluoroscopy, such as perforation during transseptal puncture. The results and recurrence rates of AF were also noted to be consistent with those of conventional AF ablation. Some operators may believe there would be an increase in procedure time with the use of zero fluoroscopic imaging; however, the procedure times were actually comparable and even shorter among the zero fluoroscopy cases than the minimal fluoroscopy ones. These results suggest that the efficiency of the electrophysiology (EP) laboratory, albeit in very experienced centers, is not affected negatively.

Low or zero fluoroscopy offers the obvious benefit of reduced exposure to radiation. This benefit is less of an issue to the patient now than in earlier days of RF ablation when fluoroscopy times could exceed one hour.^3^ Even with fluoroscopy use, the EP community has learned to minimize exposure with low frame rates, proper lead apron use, and reductions in the area of exposure. However, the cumulative exposure to the electrophysiologist and staff remains a long-term concern. If ablation procedures without radiation become the norm, there is no denying the important advantage of embracing zero-fluoroscopy methods. While deterministic effects of radiation such as cataracts or skin reactions are more commonly seen, the stochastic effect of the malignancy risk is increasingly recognized.^7^ In addition, the reduction in the use of lead aprons offers a clear benefit to the physical wellbeing of EP staff with potentially fewer chronic orthopedic issues resulting from their wear. While this advantage is less quantifiable in numbers, it can potentially extend the careers of some professionals. Regardless, zero fluoroscopy has obvious benefits to the physician and laboratory staff and, of course, the patient.

With the advent of contact-force RF catheters and multipoint mapping catheters, the physician can create highly accurate chamber geometry with confidence while also reducing RF application times. EAM and ICE also enable the visualization of structures not typically appreciated on fluoroscopy. As demonstrated in the study by Zei et al., this includes visualization of the esophagus. Similar approaches have been used for mapping the phrenic nerve to allow for pacing to be performed during ablation to mitigate the risk of injury. Therefore, the use of fluoroscopic catheter visualization has become redundant with the advent of robust EAM techniques.

The limitations of this study must also be considered. The small study size and level of center operator experience should be considered before generalizing these results. However, these results could be easily applied to operators with “very-low” fluoroscopy experience, essentially just adding in the transseptal protocol to the total scope of the procedure. In addition, as AF ablation lesion sets vary from center to center, it is hard to adopt these results to support more extensive ablation procedures other than vein isolation. Many operators use tip-deflectable sheaths to aid with positioning that are not currently visualizable on conventional mapping systems. However, new technology is already emerging that allows for such visualization, enabling the operator to adjust the position of the ablation within the sheath. The narrative of the study describes the use of this technique in patients with intracardiac lead-based devices. This could be risky in cases with relatively new leads or leads prone to dislodgement (e.g., left ventricular coronary sinus leads—applicable to the heart failure population that potentially shows the greatest benefit from ablation). In these cases, fluoroscopy can be blocked after safely achieving transseptal placement of the sheath(s). Finally, patients do not come with uniform anatomy; rarely, a patient may present with peripheral venous occlusion or tortuosity that repeatedly directs the catheter or wire down an unwanted path or they might forget to declare an inferior vena cava filter placement occurring a decade ago. These issues are easily identified with fluoroscopy but become an enigma during the access or advancement of catheters. A review with a questionnaire may help to avoid some of these issues but may not eliminate the chance of conversion of a potentially zero-fluoroscopic case to one involving fluoroscopy.

Emerging data have shown low or zero fluoroscopy is safe, effective, durable, and does not add a significant burden to the EP laboratory times or training of the staff. In addition, the data presented by Zei et al. demonstrate that it is possible to move to a completely fluoroless ablation protocol for AF. Rather than considering it an alternate modality, the EP community should now engage in a conversation of making it the new standard method. This is particularly true in the fellowship environment, as we hopefully move toward training a new generation of proceduralists that are free of the concerns of long-term radiation exposure or the necessity for lead protection.
